# STMN1–IGFBP5 axis induces senescence and extracellular matrix degradation in nucleus pulposus cells: In vivo and in vitro insights

**DOI:** 10.1186/s10020-025-01220-7

**Published:** 2025-05-03

**Authors:** Lei Li, Guangzhi Zhang, Zhili Yang, Zhenyu Cao, Dongxin Wang, Xuewen Kang

**Affiliations:** 1 Department of Orthopedics, Lanzhou University Second Hospital, Lanzhou, Gansu 730000 P.R. China; 2 The Second Clinical Medical College, Lanzhou University, Lanzhou, Gansu 730000 P.R. China; 3 Key Laboratory of Orthopedics Disease of Gansu Province, Lanzhou University Second Hospital, Lanzhou, Gansu Province 730030 P.R. China; 4 The International Cooperation Base of Gansu Province for The Pain Research in Spinal Disorders, Lanzhou, Gansu Province 730030 P.R. China

**Keywords:** STMN1, IGFBP5, Nucleus pulposus, Senescence, Extracellular matrix degradation

## Abstract

Cellular dysfunction induced by senescent nucleus pulposus (NP) cells is a key factor in the pathogenesis of intervertebral disc degeneration (IDD). Stathmin 1 (STMN1) has been proposed as a telomere-associated senescence marker implicated in senescence in many age-related diseases. Nevertheless, its role in NP cell senescence remains unclear. This study revealed that STMN1 was significantly upregulated in human degenerative and naturally aged rat NP tissue specimens. In vitro models demonstrated that STMN1 expression levels were elevated in replicative and TNF-α-induced NP senescence models. Lentiviral knockdown of STMN1 inhibited NP cell senescence, while overexpression promoted NP cell senescence, along with extracellular matrix (ECM) degradation. An in-depth mechanism indicated that insulin-like growth factor-binding protein 5 (IGFBP5), a downstream pro-senescence gene of STMN1, can induce NP cellular senescence and ECM degradation following its upregulation by STMN1. Furthermore, STMN1 knockdown reduced IGFBP5 expression and mitigated IDD development in a rat model of caudal discs puncture-induced IDD. Combined with the abovementioned results, we demonstrated for the first time that the STMN1–IGFBP5 axis can induce NP cell senescence and ECM degradation, thereby accelerating IDD development. This provides a robust foundation for the development of molecular-targeted therapies for IDD.

**Supplementary Information:**

The online version contains supplementary material available at 10.1186/s10020-025-01220-7.

## Introduction

According to statistics, about one-third of the world's musculoskeletal disorders are caused by low back pain (LBP), and its disability rate is increasing year by year, gradually becoming the leading cause of disability in underdeveloped regions (Cieza et al. 2021; Sharma and McAuley 2022). Intervertebral disc degeneration (IDD) is the primary cause of LBP (Jiang et al. 2023). However, the pathogenesis of IDD is complex, and it lacks effective treatments. Its pathogenesis is related to various factors, including the production of senescent cells, abnormal mechanical stimuli, metabolic disorders, inflammatory responses, and oxidative stress (Silwal et al. 2023; Francisco et al. 2022; Wang et al. 2023). Therefore, an in-depth understanding of the molecular mechanisms underlying IDD pathogenesis will provide new therapeutic strategies.

The intervertebral disc (IVD) provides flexibility and shock absorption within the spine and consists of a gel-like nucleus pulposus (NP) at the center, an external annulus fibrosus (AF), and a cartilaginous endplate connecting the upper and lower vertebrae. The NP distributes compressive forces to the surrounding AF and acts as a shock absorber, while the AF controls tensile forces during spinal flexion and extension (Patil et al. 2018). During aging, AF loses its characteristic fibrous lamellar network, while NP undergoes fibrotic changes due to the loss of proteoglycans in the extracellular matrix (ECM) and subsequent water loss. As a result, AF rupture in older patients with IDD is associated with a severely reduced IVD (Wang et al. 2016). However, NP cell senescence and disturbances in ECM metabolism are often considered key components in the development of IDD (Xu et al. 2023). Senescent NP cells affect the function of surrounding healthy NP cells and increase the release of intracellular inflammatory factors and catabolism of the ECM. Disturbances in ECM catabolism and anabolism accelerate IDD (Mohd Isa et al. 2022). Additionally, numerous studies have reported protective effects against IDD by inhibiting NP cell senescence and ECM degradation (Zhang et al. 2022; Chen et al. 2018). Therefore, studies on the mechanisms of NP cell senescence and ECM metabolism may help better understand the pathogenesis of IDD and identify new potential therapeutic targets.

Stathmin 1 (STMN1) is an important microtubule-binding protein that regulates microtubule dynamics. STMN1 can bind to α/β microtubule protein heterodimers, resulting in the inhibition of microtubule assembly or promotion of microtubule dissociation (Rubin and Atweh 2004). STMN1 is highly expressed in many tumor cell types and promotes cell proliferation, migration, and invasion (Karakhan and Sokolinskii 1986; Wieland 1981). However, in non-tumor cells, STMN1 is considered a telomere-associated senescence marker, and its expression increases in human senescent cells with telomere dysfunction or DNA damage (Jiang et al. 2008). Similarly, STMN1 is an age-related gene that is highly expressed in the brain tissue of patients with Alzheimer’s disease (Maunoury et al. 1992). Thus, STMN1 plays an important role in the cellular aging process of cells. However, its role in the development of IDD remains unclear. Furthermore, the identification of STMN1 as a significantly differentiated protein in the pathogenesis of IDD in our previously published proteomic analysis of senescent human NP tissues (Zhang et al. 2023) necessitated further research.

Therefore, this study aimed to clarify the expression of STMN1 in IDD and its potential role and molecular mechanism in NP cell senescence and ECM metabolism using in vivo and in vitro experiments. The downstream pro-senescence gene of STMN1, insulin-like growth factor-binding protein 5 (IGFBP5), was screened and identified by PCR-Array technology. The activation of the STMN1-IGFBP5 axis was shown to promote NP cell senescence and ECM degradation, providing a good working foundation for refining molecularly targeted therapy for IDD.

## Materials and methods

### Antibodies and reagents

The following antibodies were used for this study: STMN1 (Abcam, Cambridge, UK), p16 (ImmunoWay, California, USA; Abcam, Cambridge, UK), P21, MMP3, ADAMTS4, Collagen II, and Aggrecan (Affinity, Zhenjiang, China), and IGFBP5 (Abmart, Shanghai, China; ABclonal, Wuhan, China). The β-actin antibody was obtained from ZSGB-Bio (Beijing, China). Lentiviral transfection kits for STMN1 knockdown (LV-shSTMN1) and overexpression (LV-STMN1) were purchased from GeneChem (Shanghai, China). A small interfering RNA-IGFBP5 (si-IGFBP5) and a plasmid overexpressing IGFBP5 (OE-IGFBP5) were obtained from GenePharma (Shanghai, China). Lipo8000™ transfection reagent was obtained from Beyotime (Shanghai, China). Recombinant rat TNF-α reagent was obtained from PeproTech (New Jersey, USA). DMEM/F-12 cell culture media and fetal bovine serum (FBS) were obtained from Thermo Fisher Scientific (MA, USA).

### Human NP tissue samples

Twenty NP tissues were collected from the surgical specimens of patients with idiopathic scoliosis, lumbar spinal stenosis, lumbar disc herniation, and lumbar spondylolisthesis. Written informed consent was obtained from all patients. The specimens were standardized for collection according to the Pfirrmann classification (Pfirrmann et al. 1976). Low-grade NP tissues were collected from 10 patients (grades I and II, four males and six females, age 10–18 years, mean age 14.5 years) and High-grade NP tissues from 10 patients (grades IV and V, five males and five females, age 40–65 years, mean age 55.9 years) (Table S1). Exclusion criteria included infectious diseases, tumors, and surgical ineligibility.

### Experimental animal

Male Sprague–Dawley rats, weighing 180–200 g that met SPF-grade health standards were obtained from the Laboratory Animal Center of Lanzhou University.

### Extraction and culture of rat NP cells

Rat caudal vertebral NP primary cells were extracted aseptically through the following procedures: (1) sterilization; (2) NP tissue extraction; (3) digestion of NP tissues with type II collagenase (Proteintech, Wuhan, China); and (4) NP cell rest. Cell attachment was closely monitored, and fresh complete medium (DMEM/F-12 containing 11% FBS) was changed regularly. When NP cell density exceeded 90% in the cell flasks, passaging was performed.

### Cell transfection

Lentiviral transfection: When the growth density of NP cells was 50%, 10 μL of the corresponding viral stock solution was added to different groups, and 100 μL of the transfection enhancement solution P was added and mixed thoroughly to enhance lentiviral transfection. After 24 h, transfection efficiency was determined by the intensity of green fluorescence under a fluorescence microscope (Olympus, Tokyo, Japan), and knockdown efficiency was verified by western blotting.

Small interfering RNA (siRNA) transfection: Based on the reagents and instructions for siRNA transfection, the transfection mixture (125 μL basal medium + 100 pmol siRNA + 4 μL Lipo8000™ transfection reagent) was added in a six-well plate as an example. Proteins were extracted after 48 h, and knockdown efficiency was verified by Western blotting.

Plasmid transfection: The transfection mixture (125 μL of basal medium + 2.5 μg of plasmid DNA + 4 μL of Lipo8000™) was added to the six-well plate according to the plasmid transfection reagents and instructions. Proteins were extracted after 48 h, and the overexpression efficiency was verified by western blotting.

### Histological analysis

Fresh tissue specimens were fixed in 4% paraformaldehyde (Servicebio, Wuhan, China; rat tissue decalcified). The fixed tissues were dehydrated, embedded, and sliced on a paraffin sectioning machine at a thickness of 4 μm. The sliced tissues were subjected to HE, Safranin O/Fast Green, Alcian Blue, and Masson staining (Solarbio, Beijing, China). Immunohistochemistry was performed using the UltraSensitiveTM SP (Mouse/Rabbit) IHC Kit (Maxim, Fuzhou, China). Concentrations of primary antibodies were as follows: STMN1 (1:150), p16 (1:100), p21 (1:150), Collagen II (1:150), MMP3 (1:100), and IGFBP5 (1:150). After sealing, the sections were observed and photographed under a microscope (Olympus, Tokyo, Japan).

### Western blot

NP tissues or cells were lysed using RIPA lysis solution (Beyotime, Shanghai, China) at 4 °C, and total proteins were extracted. The concentrations of the extracted proteins were determined using a Bradford kit (Beyotime, Shanghai, China). Next, 4X sample loading buffer (Solarbio, Beijing, China) was added, boiled, and stored at −80 °C for use. Sodium dodecyl sulfate–polyacrylamide gel electrophoresis was used for protein separation, and proteins were electro-transferred onto polyvinylidene fluoride membranes (Millipore, MA, USA). The membranes were incubated with primary antibodies overnight at 4 °C. Concentrations of primary antibodies were as follows: Aggrecan (1:1000), ADAMTS4 (1:1000), Collagen II (1:1000), MMP3 (1:1000), p16 (1:1000), p21 (1:1000), STMN1 (1:5000), and β-actin (1:1500). We incubated the corresponding secondary antibodies at 4 °C for 1.5 h. Concentrations of secondary antibodies were as follows: anti-mouse IgG (1:5000) and anti-rabbit IgG (1:5000). The extra-ultrasensitive ECL luminous solution (Biosharp, Hefei, China) was evenly dripped onto the membrane, and the color was developed using a gel imaging system (Bio-Rad, California, USA). Protein gray values were detected using ImageJ software (National Institutes of Health, Bethesda, MD, USA).

### Senescence-associated β-galactosidase staining for cell senescence determination

NP cells were pre-inoculated onto six-well plates lined with 20 × 20 mm sterile coverslips and subjected to different interventions. When the cell growth density was about 80%, cells were stained using a senescence-associated β-galactosidase (SA-β-gal) staining Kit (Beyotime, Shanghai, China).

### Immunofluorescence

NP cells were fixed with 4% paraformaldehyde and subsequently permeabilized by adding prepared 0.25% Triton X-100 solution (Beyotime, Shanghai, China) for 0.25 h. Goat serum (10%) was added and incubated in a thermostatic water bath at 37 °C for 1 h. Incubation was carried out with the primary antibody solution at 4 °C overnight. The concentrations of primary antibodies used were as follows: STMN1 (1:150), p16 (1:150), p21 (1:150), Collagen II (1:150), MMP3 (1:150), and IGFBP5 (1:200). Secondary antibody solution was added and incubated in a constant temperature water bath at 37 °C, protected from light, for 1 h. The films were sealed with an Antifade Mounting Medium containing DAPI (Solarbio, Beijing, China), and images were observed and photographed by adjusting the field of view at different magnifications under a fluorescence microscope.

### Cellular senescence PCR-Array

The assay was performed using the Cellular Senescence PCR Array-Rat kit (WcGene, Shanghai, China) according to the manufacturer's instructions. First, RNA extraction and reverse transcription were performed on the intervening NP cells of the LV-shSTMN1-NC, LV-shSTMN1, LV-STMN1-NC, and LV-STMN1 groups to obtain cDNA. 920 μL of mixing solution was prepared (Wcgene® mRNA qPCR mix 2 × 510 μL, cDNA sample 100). The mixture was mixed well and added to the plate at 9 μL per well. Then, the plate was sealed with a transparent sealing film, and the quantitative reverse transcription polymerase chain reaction (qRT-PCR) was performed on a LightCycler® 480 II real-time fluorescence quantitative PCR instrument (Roche, Basel, Switzerland).

### Animal model and imaging assays

Following previous modeling methods reported in the literature (Elmounedi et al. 2022) and the experience accumulated by the group, we established an IDD model by performing needle puncture of the caudal discs of rats. Rats were randomly divided into four groups: Sham, IDD, IDD + NC, and IDD + LV-shSTMN1. The caudal vertebral 7/8 IVD space was located, and the skin and muscle tissues were incised using a sterile disposable surgical blade to expose the white AF tissue. A 21G needle (the selection of needle gauge was based on literature reports (Elmounedi et al. 2022; Zhang et al. 2011) and pre-experimental verification) was then inserted vertically into the NP tissue to a depth of approximately 5 mm. The needle was rotated clockwise by 360° and held in place for 30 s with micro-syringe injection of the corresponding lentivirus. After 8 weeks, the rats were anesthetized and underwent imaging evaluation using an X-ray machine (Siemens, Erlangen, Germany) and a 3.0-T Magnetic resonance imaging (MRI) machine (Siemens, Erlangen, Germany).

### Statistical analysis

All experiments in this study were independently repeated at least three times. All data conformed to a normal distribution and homogeneity of variance and are expressed as mean ± SD (mean ± SEM was used for some tissue specimen data). All experimental results were analyzed and processed, and statistical graphs were generated using the GraphPad Prism 8.0.2 software. Data between two groups were compared using an independent sample t-test, and data between three or more groups were compared using one-way ANOVA. *, **, and *** was used to indicate P < 0.05, P < 0.01, and P < 0.001, respectively; statistical significance was determined at P < 0.05.

## Results

### STMN1 expression in human degenerative NP tissues

**Fig. 1:**
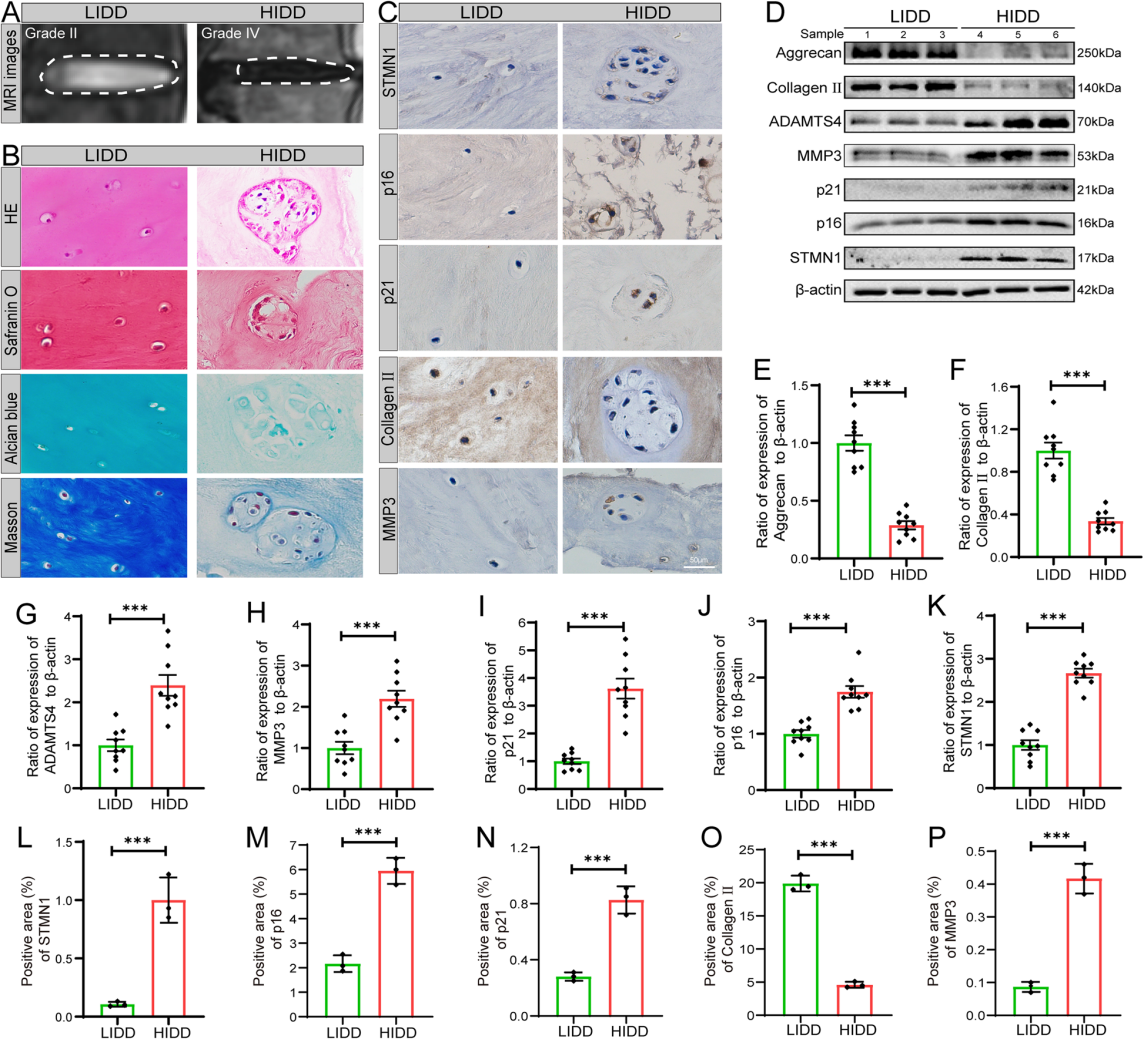
Increased STMN1 expression in human degenerative NP tissues. **A** MRI T2-weighted sagittal images of the spine in low-grade IDD (LIDD) and high-grade IDD (HIDD) groups under the Pfirrmann grading method. **B** Histological staining of NP tissue using HE, Safranin O, Alcian Blue, and Masson. **C** Immunohistochemistry showing markers of cellular senescence (p16 and p21), ECM metabolism (MMP3 and Collagen II), and STMN1 in LIDD and HIDD groups (*n* = 3). **D** Western blot showing protein expression of p16, p21, MMP3, ADAMTS4, Aggrecan, Collagen II, and STMN1 in NP tissues of LIDD and HIDD groups (*n* = 9). **E**–**K** Statistical analysis of western blot data. **L**–**P** Statistical analysis of immunohistochemical data. *P < 0.05, **P < 0.01, ***P < 0.001

We investigated the potential correlation between the expression level of STMN1 and the development of human IDD. Human NP tissue specimens collected in the clinic were grouped according to the Pfirrmann grading system: the low-grade IDD (LIDD) group (grades I and II) and the high-grade IDD (HIDD) group (grades IV and V). MRI imaging showed a significantly reduced IVD height and a significantly decreased gray level of the NP tissue in the HIDD group compared with the LIDD group (Fig. 1A). Histological analysis revealed disorganized collagen fibers of the NP tissue and a significant loss of these fibers in the HIDD group compared with the LIDD group. Additionally, NP cells clustered and formed large vacuolar cells, accompanied by a notable decrease in the surrounding proteoglycan content (Fig. 1B). Immunohistochemical analysis showed that the expression of p16, p21, MMP3, and STMN1 increased, while the expression of Collagen II decreased in the HIDD group (Fig. 1C and L–P). The protein expression level of STMN1 increased in high-grade NP tissues and correlated with alterations in cell senescence markers (p16 and p21) and ECM metabolism markers (Aggrecan, Collagen II, ADAMTS4, and MMP3) (Fig. 1D–K).

### High STMN1 expression in naturally aged rat NP cells

**Fig. 2:**
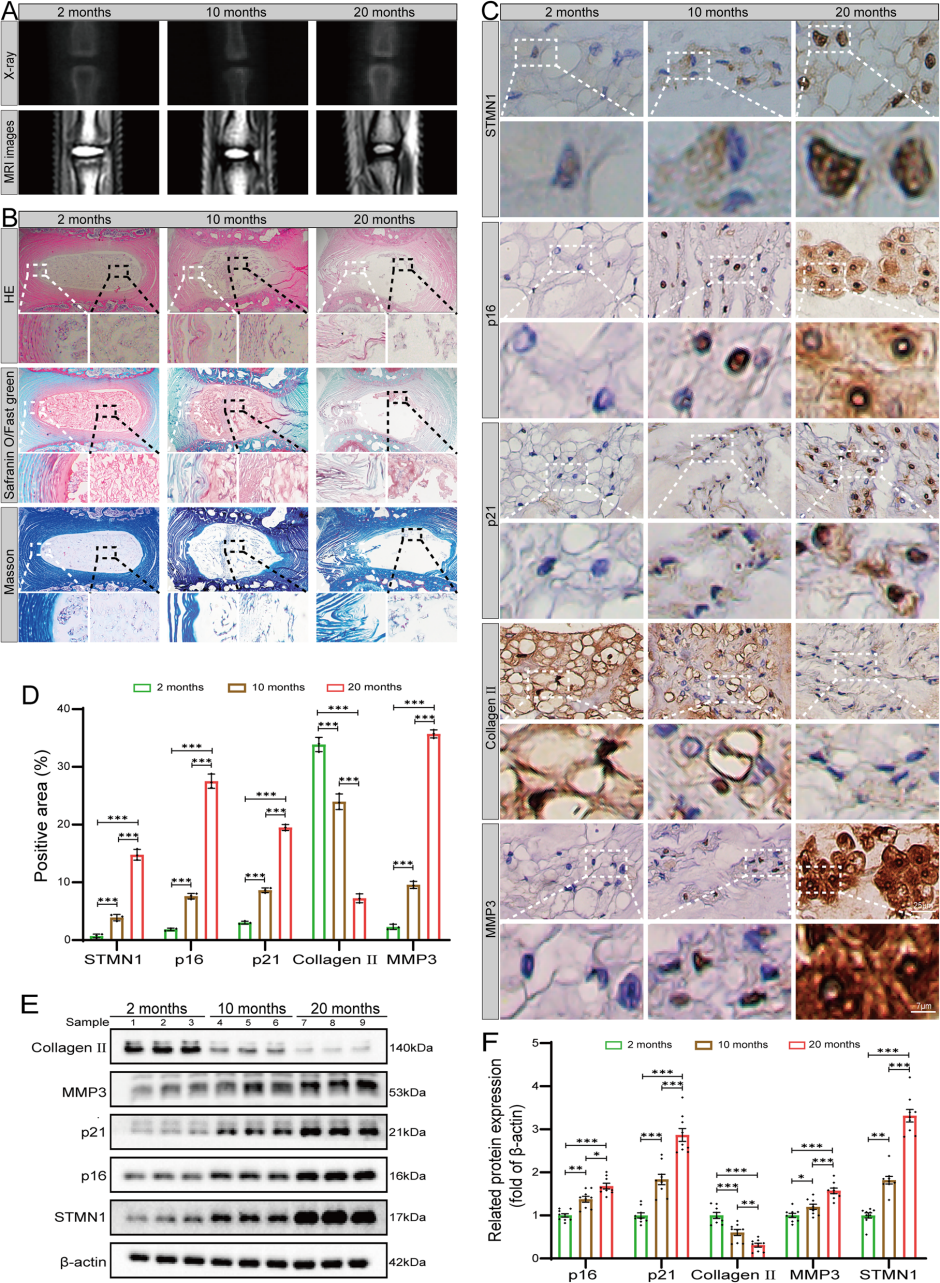
Elevated STMN1 expression in NP tissues of naturally aged rats. **A** Sagittal X-ray and MRI T2-weighted images of tailbone IVD in 2, 10, and 20 months rats. **B** HE, Safranine O/Fast green, and Masson staining images of rat NP tissues. **C** Immunohistochemical analysis of the expression of p16, p21, MMP3, Collagen II, and STMN1 in the NP of rats of different age groups. **D** Statistical analysis of immunohistochemical data (*n* = 3). **E** Western blot analysis showing protein expression of p16, p21, MMP3, Collagen, II, and STMN1 in the NP of rats of different age groups. **F** Statistical analysis of western blotting (*n* = 9). *P < 0.05, **P < 0.01, ***P < 0.001

We assessed STMN1 expression in NP cells of naturally aged rats by culturing them at different ages (2, 10, and 20 months). Sagittal X-ray and MRI T2-weighted images showed that the intervertebral space of the rat caudal vertebrae decreased as the age of the rats increased, the percentage of NP tissues in the intervertebral space decreased, and the gray value of NP tissues gradually decreased (Fig. 2A). HE, Safranine O/Fast green, and Masson staining of IVD tissues from rats of different age groups showed that with an increase in age, the NP tissues within the IVD decreased, the structural organization of the AF was disorganized, and the boundaries between the NP and AF tissues were unclear. The proteoglycan and collagen contents in NP tissues tended to decrease with age (Fig. 2B). Furthermore, there was positive correlation between imaging check and histological staining. These results indicated that our model of NP tissue degeneration in naturally aged rats was successful. Immunohistochemistry analysis showed that the expression of p16, p21, MMP3, and STMN1 increased with age and was significantly higher in NP cells of 20-month-old rats, while the expression of Collagen II showed a decreasing trend (Fig. 2C–D). Western blotting also verified the changes in these markers (Fig. 2E–F). Therefore, in the above experimental results, we confirmed NP cell senescence and ECM metabolism in naturally aged rats using imaging, histology, and related protein expression detection and verified that STMN1 was highly expressed in naturally aged rats.

### Elevated STMN1 expression in replicative and TNF-α-induced NP cell senescence models

**Fig. 3:**
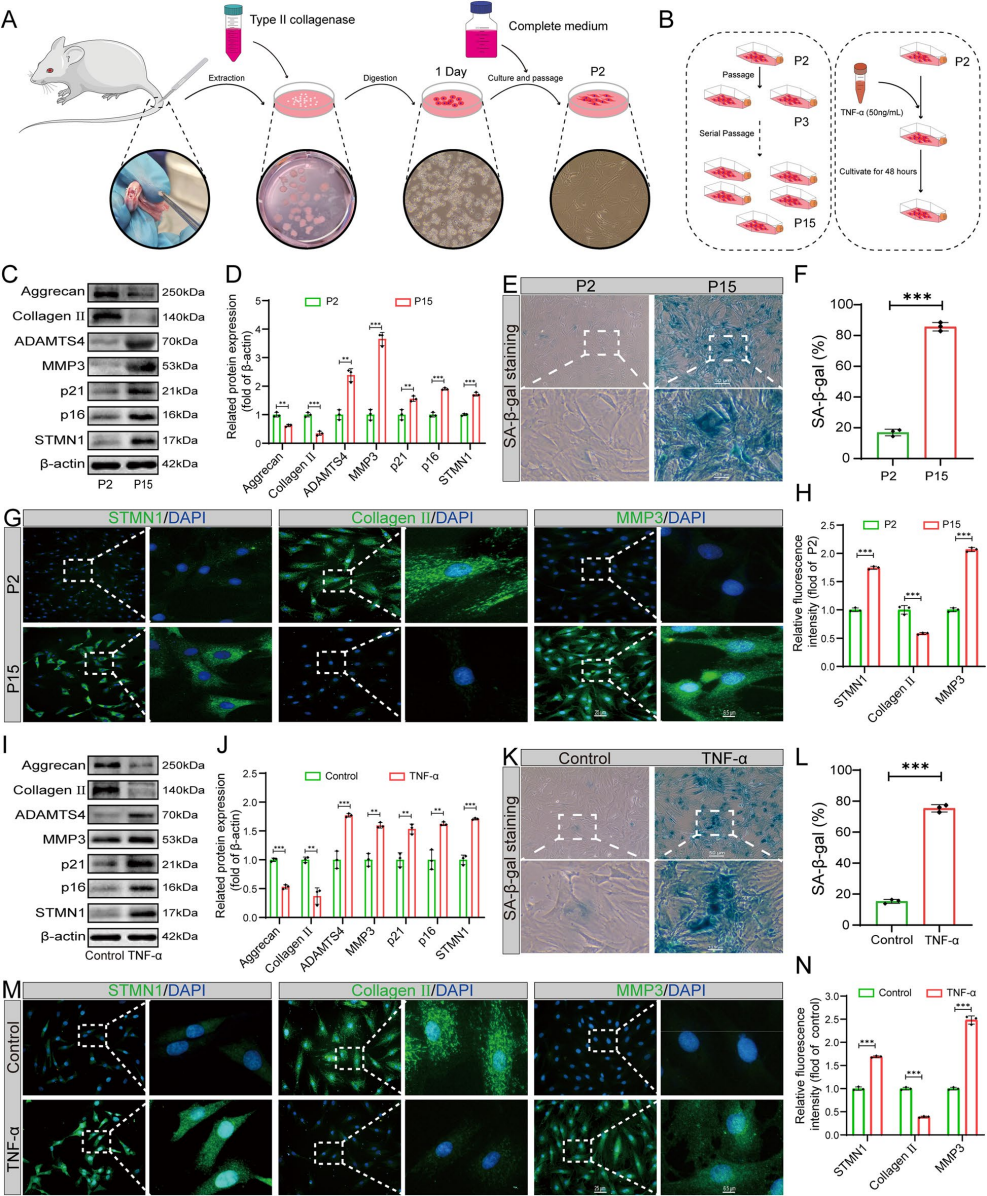
Elevated STMN1 expression in replicative and TNF-α-induced NP cell senescence models. **A** Primary rat NP cell extraction and culture. **B** Replicative and TNF-α (50 ng/mL)-induced NP cell senescence models. **C** Western blot analysis showing p16, p21, Aggrecan, Collagen II, ADAMTS4, MMP3, and STMN1 protein expression in P2 and P15 cells. **D** Statistical analysis of western blotting in (**C**) (*n* = 3); (**E**) SA-β-gal staining of P2 and P15 generation NP cells. **F** Quantitative analysis of SA-β-gal staining in (**E**) (*n* = 3). **G** Immunofluorescence analysis of STMN1, Collagen II, and MMP3 in P2 and P15 generation of NP cells. **H** Statistical analysis of immunofluorescence in (**G**) (*n* = 3). **I** Western blot analysis showing p16, p21, Aggrecan, Collagen II, ADAMTS4, MMP3, and STMN1 expression in Control and TNF-α groups. (**J**) Statistical analysis of western blotting in (**I**) (*n* = 3). **K** SA-β-gal staining of the Control and TNF-α treated NP cells. **L** Quantitative analysis of SA-β-gal staining in (K) (*n* = 3). **M** Immunofluorescence analysis of STMN1, Collagen II, and MMP3 in Control and TNF-α-treated NP cells. **N** Statistical analysis of immunofluorescence in (M) (*n* = 3). *, **, and *** indicate *P* < 0.05, *P* < 0.01, and *P* < 0.001, respectively

Through the above experiments, we initially determined that STMN1 was specifically correlated with NP cell senescence; however, the mechanisms behind its involvement in the development of cellular senescence remains unclear. Therefore, we conducted in vitro cellular experiments to further explore the specific mechanisms of action. In the resulting cellular experiments, we first verified the expression of STMN1 in senescent rat-derived primary NP cells by two commonly used cellular senescence models (replicative and TNF-α-induced NP cell senescence models). Replicative senescence is an essential part of the cellular senescence process and is a reliable model of cellular senescence (Mohamad Kamal et al. 2020). We performed comparative experiments by culturing and passaging primary rat NP cells from passage 2 (P2) to 15 (P15) (Fig. 3A–B). Compared to the P2 generation, the expression of p16, p21, MMP3, ADAMTS4, and STMN1 proteins increased in the P15 generation cells, and the expression of Aggrecan and Collagen II significantly decreased (Fig. 3C–D). SA-β-gal staining showed that the positive number of senescent NP cells increased significantly in the P15 generation (Fig. 3E–F). Immunofluorescence verified that the expression of STMN1 was elevated in senescent NP cells and was accompanied by altered ECM metabolic markers (Fig. 3G–H). These results indicate that our replicative senescence model, simulated by successive passaging of NP cells, successfully validated the expression of STMN1.

Senescence of NP cells is associated with various proinflammatory cytokines (Li et al. 2023a). TNF-α is a typical proinflammatory cytokine that has been widely used in models of NP cell degeneration. According to previous research, TNF-α at a concentration of 50 ng/mL in NP cells for 48 h induced the development of IDD (Pfirrmann, et al. 1976). Therefore, we tested this concentration and verified its reliability (Fig. 3B). Compared with the Control group, the protein expression of p16, p21, MMP3, ADAMTS4, and STMN1 increased in the TNF-α group, and the expression of Aggrecan and Collagen II significantly decreased (Fig. 3I–J). SA-β-gal staining showed a significant increase in positive senescent cells in NP cells under TNF-α intervention (Fig. 3–L). Immunofluorescence showed increased fluorescence intensity of STMN1 and MMP3 after TNF-α treatment, while Collagen II fluorescence was diminished. (Fig. 3M–N). The above results indicated that TNF-α successfully induced NP cell senescence and promoted STMN1 expression at this concentration while affecting the metabolic homeostasis of ECM.

### Effect of STMN1 knockdown and overexpression on NP cell senescence and ECM degradation

The expression of STMN1 was verified by establishing two commonly used cellular senescence models. However, whether STMN1 promotes or inhibits NP cell senescence and ECM metabolism remains unclear. To further determine the effect of STMN1 on senescence and ECM metabolism in NP cells, the specific mechanism of action was investigated using lentiviral knockdown and overexpression of STMN1 in NP cells at the gene level.

**Fig. 4:**
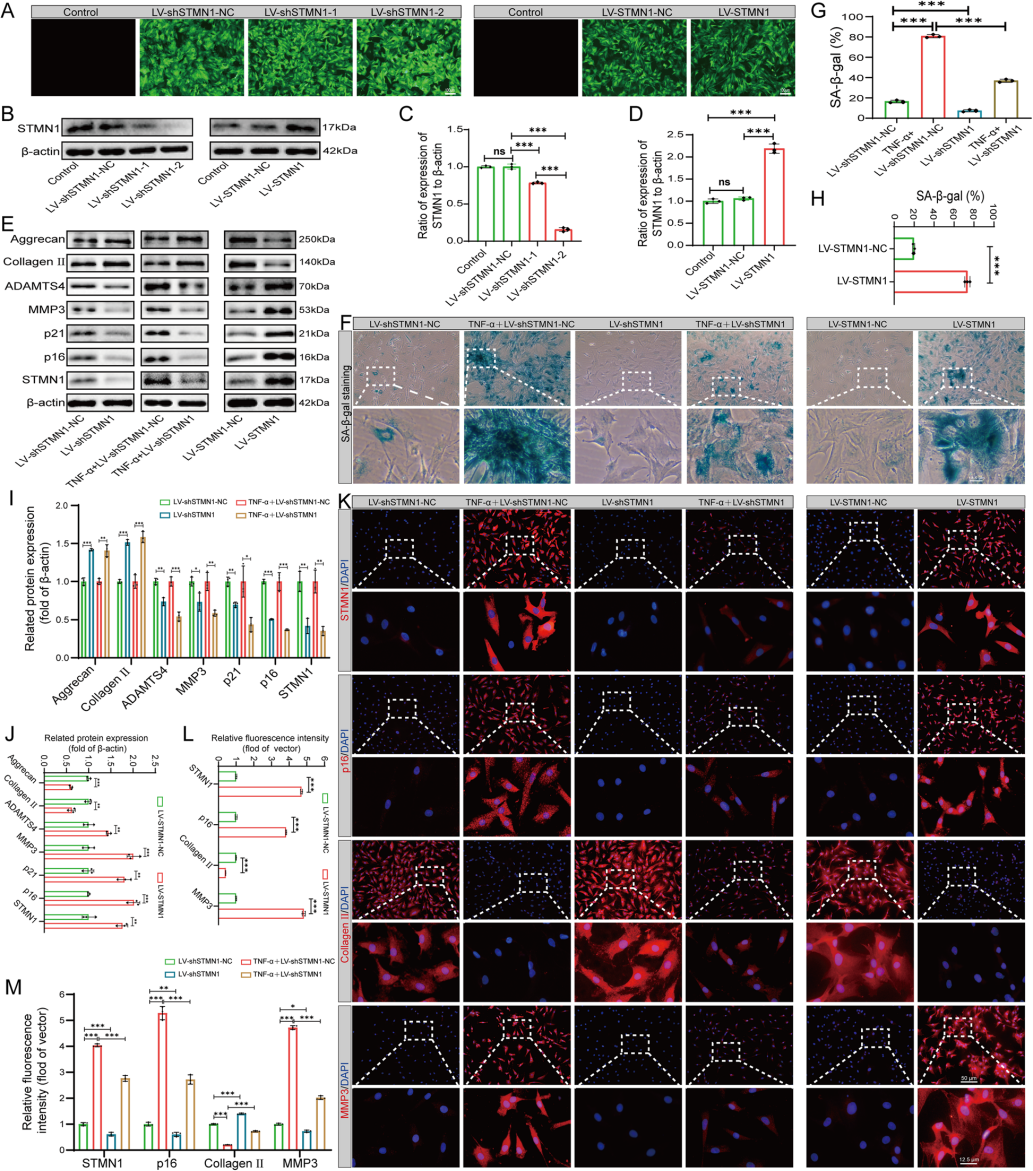
Effects of STMN1 knockdown and overexpression on NP cell senescence and ECM. **A** Green fluorescence intensity after lentiviral STMN1 knockdown (LV-shSTMN1) and overexpression (LV-STMN1). **B** Western blot analysis of STMN1 protein expression after LV-shSTMN1 and LV-STMN1 transfection into NP cells. **C**–**D** Statistical analysis of western blotting in (**B**) (*n* = 3). **E** Western blot analysis showing p16, p21, Aggrecan, Collagen II, ADAMTS4, MMP3, and STMN1 protein expression in different intervention groups. **F** NP cell SA-β-gal staining. **G**–**H** Quantitative analysis of SA-β-gal staining in (**F**) (*n* = 3). **I**–**J** Statistical analysis of western blotting in (**E**) (*n* = 3). **K** Immunofluorescence analysis of STMN1, p16, Collagen II, and MMP3 in different intervention groups. **L**–**M** Statistical analysis of immunofluorescence in (**K**) (*n* = 3). *, **, and *** indicate *P* < 0.05, *P* < 0.01, and *P* < 0.001, respectively

After 24 h of NP cell transfection, the knockdown (LV-shSTMN1-NC, LV-shSTMN1-1, and LV-shSTMN1-2) and overexpression groups (LV-STMN1-NC and LV-STMN1) exhibited an elevated green fluorescence intensity compared to the control group (Fig. 4A). This suggests that the transfection of NP cells was effective. Western blotting demonstrated that LV-shSTMN1-NC and LV-STMN1-NC had minimal effect on STMN1 expression compared to the control group. LV-shSTMN1-1 and LV-shSTMN1-2 suppressed STMN1 expression, with LV-shSTMN1-2 exhibiting the highest knockdown efficiency. Conversely, overexpression of STMN1 was markedly elevated by LV-STMN1. (Fig. 4B–D). STMN1 knockdown under TNF-α intervention was performed to observe the phenotypic alterations. Compared to the LV-shSTMN1-NC group, the expression of p16, p21, MMP3, ADAMTS4, and STMN1 decreased in the LV-shSTMN1 group, and the expression of Aggrecan and Collagen II significantly increased. Compared with the TNF-α + LV-shSTMN1-NC group, the TNF-α + LV-shSTMN1 group downregulated the protein expression levels of STMN1, p16, p21, MMP3, and ADATMS4, while expression of Aggrecan and Collagen II was upregulated (Fig. 4E–I). The expression levels of STMN1, p16, p21, MMP3, and ADATMS4 were significantly elevated in the LV-STMN1 group compared with the LV-STMN1-NC group, while the expression levels of Aggrecan and Collagen II were reduced (Fig. 4E–J). SA-β-gal staining also indicated that the knockdown and overexpression of STMN1 resulted in a reduction and elevation in the positivity of senescent cells within the NP, respectively (Fig. 4F–H). Immunofluorescence analysis showed that the red fluorescence intensity of STMN1, p16, and MMP3 was significantly higher in the TNF-α + LV-shSTMN1-NC group than in the LV-shSTMN1-NC group, while Collagen II fluorescence was diminished. However, the TNF-α + LV-shSTMN1 group reversed this result. In the overexpression group, the red fluorescence intensities of STMN1, p16, and MMP3 in the LV-STMN1 group were significantly higher than those in the LV-STMN1-NC group, while the red fluorescence intensity of Collagen II was diminished (Fig. 4K–M). Based on the above experimental results and data analysis, we found that STMN1 knockdown delayed NP cell senescence and ECM degradation in degenerative NP cells. STMN1 overexpression also promoted senescence and ECM catabolism in NP cells.

### IGFBP5 as a key pro-senescence gene downstream of STMN1

**Fig. 5:**
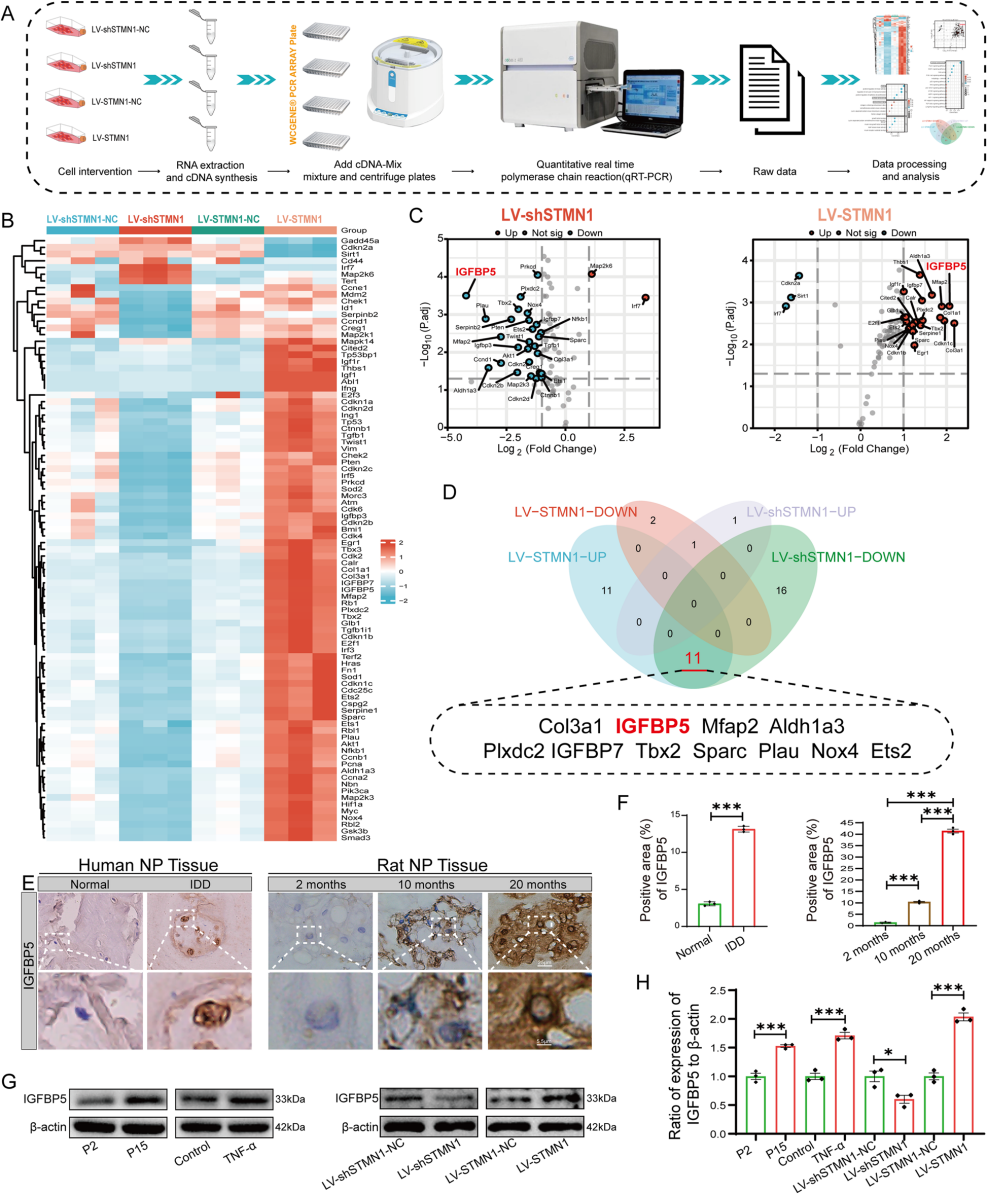
IGFBP5 as a key pro-senescence gene downstream of STMN1. **A** Sample extraction and PCR-Array data processing procedures for different interventions. **B** Heatmap of 90 cellular senescence-related genes in LV-shSTMN1 and LV-STMN1 groups. **C** Volcano plot showing differentially expressed genes of the LV-shSTMN1 group with 29 differentially expressed genes (Up: 2, Down: 27); volcano plot of the LV-STMN1 group with 25 differentially expressed genes (Up: 22, Down: 3), based on the criteria |log2 FC|≥ 1 and *p* < 0.05. **D** Venn diagram cross-tabulation analysis identifying 11 key genes involved in senescence. **E** Immunohistochemical analysis of IGFBP5 expression in human degenerative and naturally aged rat NP tissues. **F** Statistical analysis of immunohistochemistry (*n* = 3). **G** Western blot analysis showing STMN1 protein expression in different intervention groups. **H** Statistical analysis of Western blotting (*n* = 3). *, **, and *** indicate *P* < 0.05, *P* < 0.01, and *P* < 0.001, respectively

The above experiments demonstrated that STMN1 plays a role in NP cell senescence. However, the precise mechanism through which STMN1 induces senescence in NP cells remains undetermined. Therefore, determining whether STMN1 can regulate a specific downstream pro-senescence gene that causes the onset of NP cell senescence was essential. The PCR-Array technique was employed to ascertain the expression of 90 genes associated with cellular senescence in the LV-shSTMN1 and LV-STMN1 groups. Figure 5A shows the flow chart of the PCR-Array detection and processing data of cells from different intervention groups.

Heatmap analysis showed a significant reduction in the expression of most genes in the LV-shSTMN1 group compared with the LV-shSTMN1-NC group. Conversely, the expression of most genes in the LV-STMN1 group exhibited a significant increase compared to the LV-STMN1-NC group (Fig. 5B). Principal component analysis (PCA) of gene expression in different intervention groups showed that PC1 + PC2 was 96.8% in the LV-shSTMN1 group and 95.8% in the LV-STM1 group, and all the PCA results indicated a high degree of variability in gene expression with high reliability (Fig. S1 A). The volcano plot analysis of the different intervention groups revealed 29 differentially expressed genes (Up: 2, Down: 27) in the LV-shSTMN1 group and 25 differentially expressed genes (Up: 22, Down: 3) in the LV-STMN1 group, all of which were based on |log2 FC|≥ 1 and p < 0.05 as the thresholds (Fig. 5C). Functional and phenotypic enrichment analyses showed that these differentially expressed genes were closely related to cellular senescence and ECM (Fig. S1B). Therefore, to further screen the target genes, we performed a Venn diagram analysis of the screened genes in the four groups (LV-shSTMN1-Up, LV-shSTMN1-Down, LV-STMN1-Up, and LV-STMN1-Down) and identified 11 genes with alterations that followed the same trend as STMN1 overexpression and knockdown (Fig. 5D). qRT-PCR analysis showed that, among the 11 genes detected by mRNA expression, the expression of IGFBP5 was the lowest in the LV-shSTMN1 group. Conversely, the expression of IGFBP5 in the LV-STMN1 group was second only to that of Col3a1 (Fig. S1 C). Therefore, by combining the above experimental results, we identified IGFBP5 as a key pro-senescence gene downstream of STMN1. To verify whether IGFBP5 was associated with NP cell senescence, we performed immunohistochemical and western blot analyses. Immunohistochemical analysis showed increased expression of IGFBP5 in human degenerative NP tissues and NP tissues from naturally senescent rats (Fig. 5E–F). Western blot results showed elevated protein expression of IGFBP5 in the P15 generation compared with the P2 generation of NP cells. Similarly, IGFBP5 showed high expression after TNF-α treatment (NP cells were derived from P2 generation) (Fig. 5G–H). We concluded that IGFBP5, a downstream target of STMN1, may play a vital role in the senescence of NP cells.

### Effect of IGFBP5 knockdown and overexpression on NP cell senescence and ECM degradation

**Fig. 6:**
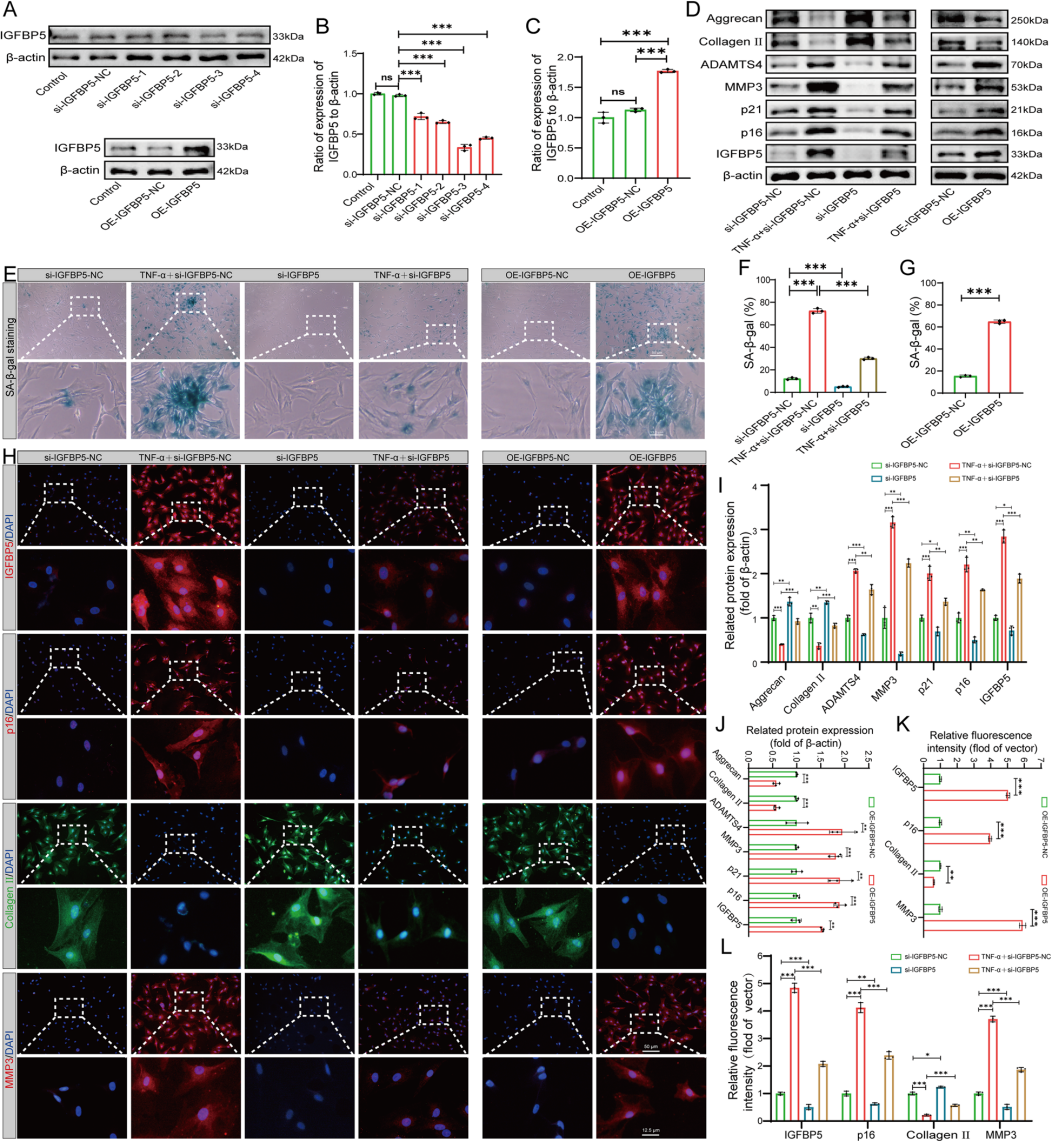
Effects of IGFBP5 knockdown and overexpression on NP cell senescence and ECM degradation (**A**) Western blot analysis showing STMN1 protein expression after si-IGFBP5 and OE-IGFBP5 transfection in NP cells. **B**–**C** Statistical analysis of western blotting in (**A**) (*n* = 3). **D** Western blot analysis showing p16, p21, Aggrecan, Collagen II, ADAMTS4, MMP3, and STMN1 protein expression in different intervention groups. **E** NP cell SA-β-gal staining. **F**–**G** Quantitative analysis of SA-β-gal staining in (**E**) (*n* = 3). **H** Immunofluorescence analysis of STMN1, p16, Collagen II, and MMP3 in different intervention groups. **I**–**J** Statistical analysis of western blotting in (**D**) (*n* = 3). **K**–**L** Histograms of statistical analysis in (**H**) (*n* = 3). *, **, and *** indicate *P* < 0.05, *P* < 0.01, and *P* < 0.001, respectively

Although the above experiments demonstrated that IGFBP5 is a crucial downstream pro-senescence-related gene of STMN1, the specific effects of IGFBP5 on senescence and ECM metabolism in NP cells remain unverified. We conducted gene-level experiments using rat-derived NP cells to further determine the role of IGFBP5 and elucidate the regulatory mechanism of STMN1. We transfected NP cells with a siRNA to knock down IGFBP5 (si-IGFBP5) and a plasmid to overexpress IGFBP5 (OE-IGFBP5) to clarify the mechanism of its action.

si-IGFBP5-NC and OE-IGFBP5-NC groups exhibited minimal effect on the expression of IGFBP5 compared with that in the Control group. Conversely, si-IGFBP5-1, si-IGFBP5-2, si-IGFBP5-3, and si-IGFBP5-4 inhibited the expression of IGFBP5, with si-IGFBP5-3 exhibiting the highest knockdown efficiency. Additionally, the overexpression of OE-IGFBP5 was evident (Fig. 6A–C). Western blot analysis revealed a reduction in the expression of IGFBP5, p16, p21, MMP3, and ADATMS4 in the si-IGFBP5 group compared with the si-IGFBP5-NC group. Conversely, increased Aggrecan and Collagen II expression were observed. Compared with the TNF-α + si-IGFBP5-NC group, the TNF-α + si-IGFBP5 group showed a downregulation of IGFBP5, p16, p21, MMP3, and ADATMS4 expression while concurrently exhibiting an upregulation of Aggrecan and Collagen II expression. The expression levels of IGFBP5, p16, p21, MMP3, and ADATMS4 were significantly higher in the OE-IGFBP5 group than in the OE-IGFBP5-NC group. Conversely, the expression levels of Aggrecan and Collagen II decreased (Fig. 6D, I, and J). SA-β-gal staining also reflected the knockdown of IGFBP5 and overexpression of IGFBP5, which decreased and elevated the positivity of senescent cells within the NP, respectively (Fig. 6E–G). Immunofluorescence analysis also verified the trend of the abovementioned groups of markers (Fig. 6H, K, and L). Overall, the above experimental results and data analysis demonstrated that IGFBP5 knockdown had an inhibitory effect on senescence and ECM degradation in NP cells, while overexpression of IGFBP5 promoted senescence and ECM catabolism in NP cells.

### STMN1 promotes NP cell senescence and ECM degradation via IGFBP5 regulation

**Fig. 7:**
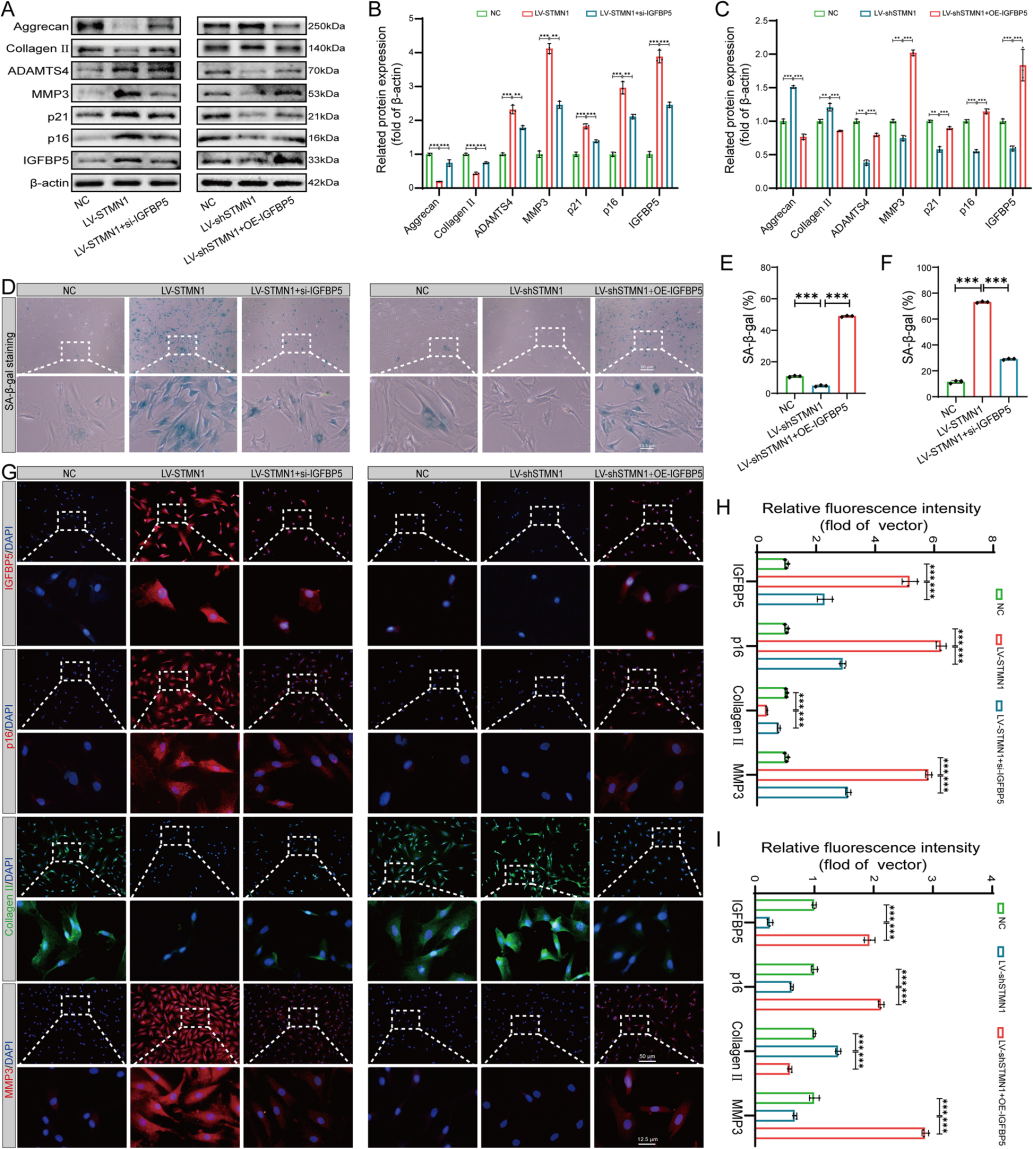
STMN1 promotes NP cell senescence and ECM degradation via IGFBP5 regulation. **A** Western blot analysis showing p16, p21, Aggrecan, Collagen II, ADAMTS4, MMP3, and STMN1 expression in different intervention groups. **B**–**C** Statistical analysis of western blotting in (**A**) (*n* = 3). **D** NP cell SA-β-gal staining. **E**–**F** Quantitative analysis of SA-β-gal staining in (**D**) (*n* = 3). **G** Immunofluorescence of STMN1, p16, Collagen II, and MMP3 in different intervention groups. **H**–**I** Statistical analysis of immunofluorescence in (**G**) (*n* = 3). *, **, and *** indicate *P* < 0.05, *P* < 0.01, and *P* < 0.001, respectively

The following experiment demonstrated that IGFBP5, a downstream gene of STMN1, can be regulated by STMN1, thereby affecting senescence and ECM metabolism levels in NP cells. The regulatory mechanism was validated using rescue experiments. Compared with the LV-STMN1 group, the LV-STMN1 + si-IGFBP5 group exhibited a reduction in the protein expression levels of ADAMTS4, MMP3, p21, p16, and IGFBP5, accompanied by an increase in the expression of Aggrecan and Collagen II to varying degrees. Conversely, the LV-shSTMN1 + OE-IGFBP5 group exhibited elevated expression levels of ADAMTS4, MMP3, p21, p16, and IGFBP5, accompanied by decreased expression of Aggrecan and Collagen II to varying degrees compared to the LV-shSTMN1 group. (Fig. 7A–C). The SA-β-gal staining showed that the LV-STMN1 + si-IGFBP5 group had partially reduced positivity rate of senescent cells in the NP compared to the LV-STMN1 group, while the LV-shSTMN1 + OE-IGFBP5 group had significantly increased positivity rate of senescent cells within the NP compared to the LV-shSTMN1 group (Fig. 7D–F). Immunofluorescence analysis also confirmed that STMN1 affected the expression of p16, collagen II, and MMP3 by regulating IGFBP5. (Fig. 7G–I). Our results demonstrated that IGFBP5, a pro-senescence gene downstream of STMN1, can be upregulated by STMN1 to promote NP cellular senescence and alter ECM metabolism.

### Knockdown of STMN1 improves the progression of IDD in the rat caudal discs puncture model

We established an in vivo rat caudal discs puncture-induced IDD model and injected LV-shSTMN1 into the caudal discs to verify whether inhibition of STMN1 expression alleviated IDD in rats.

**Fig. 8:**
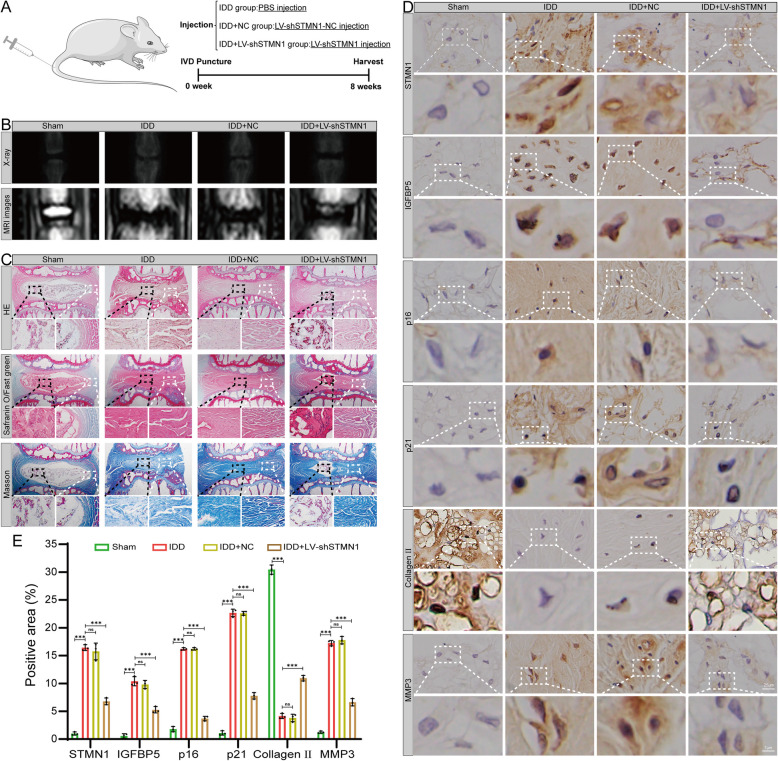
Knockdown of STMN1 improves the progression of IDD in the rat caudal discs puncture model. **A** Schematic diagram of IDD modeling in rats. **B** X-ray and MRI T2-weighted imaging images of rat caudal vertebrae in different intervention groups. **C** HE, Safranine O/Fast green, and Masson staining maps of IVD in rats in different intervention groups. **D** Immunohistochemical analysis of STMN1, p16, p21, Collagen II, and MMP3 in the NP tissue samples of rats. **E** Statistical analysis of immunohistochemistry (*n* = 3). *, **, and *** indicate *P* < 0.05, *P* < 0.01, and *P* < 0.001, respectively

As shown in Fig. 8A, the degree of IDD in rats was assessed using X-ray imaging and MRI after 8 weeks (Fig. 8B). The X-ray results showed that the intervertebral space heights in the IDD and IDD + NC groups were significantly lower compared to the Sham group, while those in the IDD + LV-shSTMN1 group improved. The MRI results showed that the IVD T2-weighted signal intensity in the IDD + LV-shSTMN1 group was higher than that in the IDD and IDD + NC groups. The HE staining results demonstrated that, compared to the Sham group, the normal structure of the NP tissue in the IDD and IDD + NC groups was significantly disrupted. A substantial quantity of AF tissues replaced a considerable loss of NP cells and ECM. The AF appeared to be fractured, and its border with the NP tissue was indistinct. Conversely, the number of NP cells increased in the IDD + LV-shSTMN1 group, the ECM of NP tissues was restored, and the boundary between the NP and AF tissues was more evident. Safranine O/Fast green and Masson staining results demonstrated a significant reduction in proteoglycans and collagen in the IDD and IDD + NC groups compared with the Sham group. Conversely, the IDD + LV-shSTMN1 group showed restoration of proteoglycans and collagen (Fig. 8C).

The immunohistochemistry results of IGFBP5 showed that the protein expression of IGFBP5 in the IDD and IDD + NC groups significantly increased compared with that in the Sham group, while the expression of IGFBP5 decreased after LV-shSTMN1 treatment, which further verified that STMN1 regulated IGFBP5 in cellular experiments. Immunohistochemical analysis of cellular senescence and ECM metabolism-related biomarkers revealed that the expression of p16, p21, and MMP3 was increased, while that of Collagen II diminished in the IDD and IDD + si-NC groups, relative to the Sham group. Conversely, the expression of these biomarkers was reversed in the LV-shSTMN1 group (Fig. 8D–E).

The above results indicate that our fine-needle puncture model of rat caudal spine IDD was successful and that increased cellular senescence accompanied by a disturbed ECM metabolic balance was observed in the NP cells of rats with IDD. Conversely, local injection of LV-shSTMN1 effectively inhibited rat NP cell senescence and ECM degradation and increased ECM anabolism. Therefore, we concluded that the knockdown of STMN1 improved the progression of IDD in the rat caudal discs puncture model.

## Discussion

Throughout the development of IDD, intrinsic pathophysiological changes in the NP play a significant role due to its unique and functional characteristics (Mohd Isa et al. 2022). Typically, from a macroscopic perspective, degeneration leads to a reduction in NP volume, a dramatic decrease in water content, and a loss of viscoelastic properties, impairing the ability of NP to withstand normal or abnormal compressive forces in the spine. From a microscopic perspective, there is a decrease in NP cell numbers and a gradual increase in senescent cells, accompanied by the production of various proinflammatory factors that disrupt the homeostasis of the internal environment of the NP (Wang et al. 2016). Therefore, an increase in the number of senescent NP cells is an important pathological factor that accelerates NP tissue degeneration. The balanced state of ECM anabolism and catabolism in healthy NP cells plays a key role in ECM stabilization (Luo et al. 2023). However, senescent NP cells secrete a variety of molecules such as common TNF-α, IL-1β, IL-6, IL-8, and MMPs. This can lead to chronic low-grade inflammation within the NP, which disrupts the balance between ECM synthesis and catabolism and promotes the transition from healthy NP cells to senescent cells (Xu et al. 2023). Therefore, it is particularly important to study the senescence and ECM metabolism of NP cells and explore the intrinsic regulatory mechanisms to provide a reliable scientific basis for improving the molecular treatment of IDD.

STMN1 is a ubiquitous cytoplasmic phosphoprotein characterized as a microtubule-binding protein that plays important roles in mitosis, motility, process formation, and intracellular transport (Rubin and Atweh 2004). The most common function of STMN1 is to alter microtubule stability by interacting with microtubule proteins. Crest myeloid muscular dystrophy is an age-related neurodegenerative disease caused by survival motor neuron (SMN) protein deficiency due to mutations or deletions in the SMN1 gene. Wen et al. (Wen et al. 2010) identified STMN1 in NSC34 cells screened for SMN knockdown using proteomics analysis. Abnormal upregulation of STMN1 in vitro and in vivo resulted in reduced levels of polymerized microtubule proteins. However, STMN1 knockdown repaired microtubule network defects in SMN-deficient cells, promoted axonal growth, and reduced mitochondrial transport defects in SMA-like motor neurons. Additionally, multiple studies have shown a strong association between STMN1 and age-related diseases, and it is considered a marker of cellular senescence due to telomere damage (Jiang et al. 2008; Lekva et al. 2022). However, the role of STMN1 in IDD has not yet been reported, and its involvement in NP cell senescence has attracted great interest.

Thus, we demonstrated that the expression of STMN1 is elevated in NP tissues from human degenerative clinical specimens and naturally aged rats. This suggests that STMN1 is associated with cellular senescence and ECM metabolic disorders. In the cell function experiment, we first extracted the primary NP cells of rats and cultured them, which were verified by two kinds of aging models of NP cells. We found that STMN1 was highly expressed in both replicative and TNF-α-induced aging models. Further studies showed that when LV-shSTMN1 was transfected into NP cells, the TNF-α-induced senescence and ECM metabolism of NP cells were significantly reduced. Transfection of LV-STMN1 into NP cells increased senescence and ECM metabolism. These results suggest that STMN1 promotes senescence and ECM degradation in NP cells.

Further investigation of the regulatory mechanism of STMN1 revealed that IGFBP5 may be a key gene downstream of STMN1 that promotes the senescence of NP cells. IGFBP5 is a secreted IGF-binding protein that belongs to the IGFBP family, which is a group of proteins capable of binding to IGF and has a bipartite effect on IGF-I and IGF-II (Song et al. 2022). In addition to regulating IGF activity, it has many other biological functions independent of IGF, including inflammatory response, cell adhesion, cell senescence, cell migration, and fibrosis. IGFBP5 induces the onset of cellular senescence in a variety of cells (Sanada et al. 2018; Soriano-Arroquia et al. 2016). IGFBP5 is reportedly associated with apoptosis of NP cells (Chen et al. 2019; Wang et al. 2019). However, no studies have reported whether IGFBP5 induces senescence in NP cells. Therefore, to determine whether IGFBP5 affects NP cell senescence and ECM metabolism, si-IGFBP5 and OE-IGFBP5 were transfected in TNF-α-intervened NP cells and were found to have the same effect on NP cell senescence and ECM metabolism as STMN1. We verified whether STMN1 promotes changes in NP cell senescence and ECM phenotype by influencing IGFBP5 expression. We knocked down IGFBP5 after overexpressing STMN1 in NP cells and found that the knockdown of IGFBP5 improved the effect of STMN1 on the senescence and ECM metabolic levels of NP cells. Overexpression of IGFBP5 after the knockdown of STMN1 reversed the protective effects of STMN1 knockdown. Therefore, by combining the above experimental results, we determined that STMN1 induced changes in NP cell senescence and ECM metabolism by regulating the expression of IGFBP5. In animal experiments, LV-shSTMN1 was injected into the caudal vertebral NP tissues of puncture-modelled rats, and downregulation of STMN1 expression significantly improved NP cell senescence and ECM metabolism, delaying the development of IDD in rats.

**Fig. 9:**
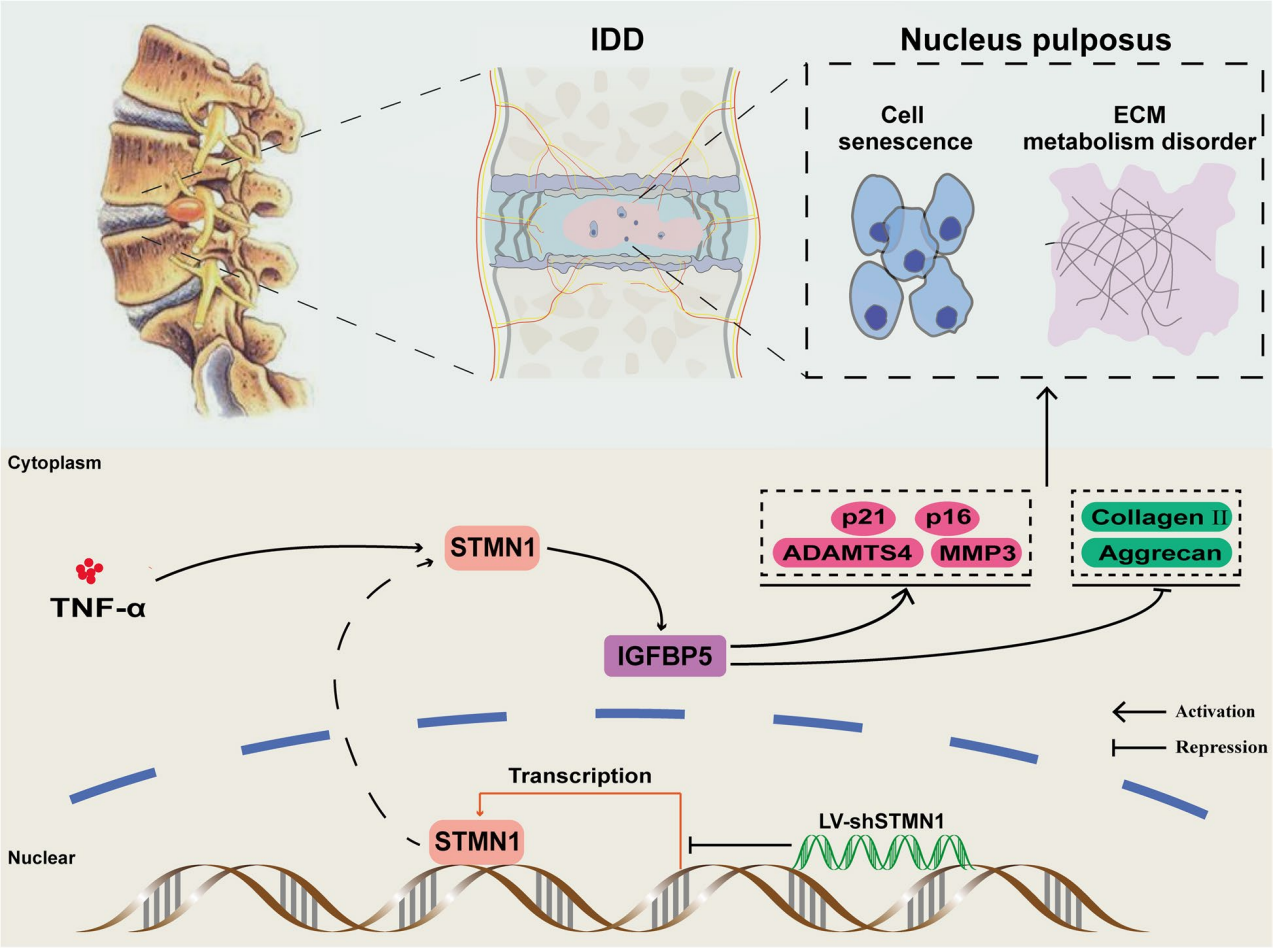
Schematic representation of the STMN1–IGFBP5 axis mediating NP cell senescence and ECM degradation STMN1 upregulated indicators of cellular senescence (p16, p21) and ECM catabolism (MMP3 and ADAMTS4) and suppressed anabolism (Aggrecan and Collagen II) by promoting the expression of IGFBP5, which mediated the acceleration of IDD progression by altered NP cellular senescence and ECM metabolism

In this study, we demonstrated for the first time that the expression level of STMN1 is elevated in human degenerative and naturally senescent rat NP tissue specimens. STMN1 was able to promote the development of IDD, and it accelerates the progression of IDD potentially by upregulating IGFBP5, thus inducing NP cell senescence and ECM degradation (Fig. 9 shows a schematic diagram of the mechanism of this experiment). However, the experiment has the following limitations: First, the experiment was not performed on human NP cells, lacking persuasive power; Second, the screening of downstream senescence-promoting gene IGFBP5 might have some drawbacks, and there may be senescence-promoting genes with a more pronounced regulatory effect on STMN1 in addition to the 90 senescence-related genes screened in the experiment. Third, in the animal experiments, the regulatory mechanism of STMN1 on IGFBP5 is not perfect. Fourth, the rat model of IDD has inherent limitations, thus requiring mechanistic validation using large animal models or non-human primates.

In the introduction, we mentioned that the predominant effect of STMN1 is the destabilization of microtubules. Li et al. (Li et al. 2022) first suggested that microtubule stabilization significantly inhibited cartilage fibrosis and increased hyaline cartilage ECM. In a subsequent study, they found that microtubule stabilization promoted cartilage regeneration in a rat model of cartilage injury by inhibiting YAP activity, suggesting that the maintenance of microtubule stability is a promising therapeutic target for the treatment of osteoarthritis and cartilage injury (Li et al. 2023). Similarly, in a study on IDD, Zhang et al. (Zhang et al. 2023) revealed for the first time that degenerative NP cells that stabilize microtubules can stimulate the Hippo signaling pathway and inhibit YAP activity to promote the expression of Collagen II, an indicator of ECM anabolism in NP. Therefore, in conjunction with the literature reports, we speculate that the stability of microtubules in NP cells is related to cellular senescence and that STMN1 can regulate microtubule stability and promote the onset of senescence and ECM metabolic changes in NP cells. These hypotheses may provide direction for future follow-up studies regarding this topic of research.

## Conclusion

Based on the results of this study, we propose for the first time that STMN1 is involved with the process of IDD and that inhibition of the STMN1–IGFBP5 axis can reduce NP cell senescence and ECM degradation, providing a potential therapeutic target for IDD treatment.

## Supplementary Information

Supplementary Material 1.

## Data Availability

No datasets were generated or analysed during the current study.

## Abbreviations

IDD: Intervertebral disc degeneration
IVD: Intervertebral disc
NP: Nucleus pulposus
AF: Annulus fibrosus
STMN1: Stathmin 1
ECM: Extracellular matrix
IGFBP5: Insulin-like growth factor-binding protein 5
TNF-α: Tumor necrosis factor-α
FBS: Fetal bovine serum
PCA: Principal component analysis
MRI: Magnetic resonance imaging
qRT-PCR: Quantitative reverse transcription polymerase chain reaction
SMN: Survival motor neuron

## References

[CR1] Chen J, et al. Sirt6 overexpression suppresses senescence and apoptosis of nucleus pulposus cells by inducing autophagy in a model of intervertebral disc degeneration. Cell Death Dis. 2018;9(2):56. 29352194 10.1038/s41419-017-0085-5 PMC5833741

[CR2] Chen Z, et al. Down-regulation of insulin-like growth factor binding protein 5 is involved in intervertebral disc degeneration via the ERK signalling pathway. J Cell Mol Med. 2019;23(9):6368–77. 31290273 10.1111/jcmm.14525 PMC6714225

[CR3] Cieza A, et al. Global estimates of the need for rehabilitation based on the Global Burden of Disease study 2019: a systematic analysis for the Global Burden of Disease Study 2019. Lancet. 2021;396(10267):2006–17. 33275908 10.1016/S0140-6736(20)32340-0 PMC7811204

[CR4] Elmounedi N, et al. Impact of Needle Size on the Onset and the Progression of Disc Degeneration in Rats. Pain Physician. 2022;25(6):509–17. 36122262

[CR5] Francisco V, et al. A new immunometabolic perspective of intervertebral disc degeneration. Nat Rev Rheumatol. 2022;18(1):47–60. 34845360 10.1038/s41584-021-00713-z

[CR6] Jiang H, et al. Proteins induced by telomere dysfunction and DNA damage represent biomarkers of human aging and disease. Proc Natl Acad Sci U S A. 2008;105(32):11299–304. 18695223 10.1073/pnas.0801457105 PMC2516278

[CR7] Jiang W, et al. Intervertebral disc human nucleus pulposus cells associated with back pain trigger neurite outgrowth in vitro and pain behaviors in rats. Sci Transl Med. 2023;15(725):eadg7020. 38055799 10.1126/scitranslmed.adg7020 PMC12083434

[CR8] Karakhan VB, Sokolinskii AV. Spinal endoscopy. Zh Vopr Neirokhir Im N N Burdenko. 1986;3:48–51. 3526768

[CR9] Lekva T, et al. Markers of cellular senescence is associated with persistent pulmonary pathology after COVID-19 infection. Infect Dis (Lond). 2022;54(12):918–23. 35984738 10.1080/23744235.2022.2113135

[CR10] Li J, et al. Articular fibrocartilage-targeted therapy by microtubule stabilization. Sci Adv. 2022;8(46):eabn8420. 36399569 10.1126/sciadv.abn8420 PMC9674280

[CR11] Li Z, et al. Regulatory Effect of Inflammatory Mediators in Intervertebral Disc Degeneration. Mediators Inflamm. 2023a;2023:6210885. 37101594 10.1155/2023/6210885 PMC10125773

[CR12] Li J, et al. Microtubule stabilization targeting regenerative chondrocyte cluster for cartilage regeneration. Theranostics. 2023;13(10):3480–96. 37351173 10.7150/thno.85077 PMC10283062

[CR13] Luo ZB, et al. Achilles’ heel-The significance of maintaining microenvironmental homeostasis in the nucleus pulposus for intervertebral discs. Int J Mol Sci. 2023;24(23):16592. 38068915 10.3390/ijms242316592 PMC10706299

[CR14] Maunoury R, et al. Developmental regulation of villin gene expression in the epithelial cell lineages of mouse digestive and urogenital tracts. Development. 1992;115(3):717–28. 1425351 10.1242/dev.115.3.717

[CR15] Mohamad Kamal NS, et al. Aging of the cells: Insight into cellular senescence and detection Methods. Eur J Cell Biol. 2020;99(6):151108. 32800277 10.1016/j.ejcb.2020.151108

[CR16] Mohd Isa IL, et al. Discogenic low back pain: anatomy, pathophysiology and treatments ofintervertebral disc degeneration. Int J Mol Sci. 2022;24(1):208. 36613651 10.3390/ijms24010208 PMC9820240

[CR17] Patil P, et al. Cellular senescence in intervertebral disc aging and degeneration. Curr Mol Biol Rep. 2018;4(4):180–90. 30473991 10.1007/s40610-018-0108-8 PMC6248341

[CR18] Pfirrmann CW, et al. Magnetic resonance classification of lumbar intervertebral disc degeneration. Spine (Phila Pa 1976). 2001;26(17):1873–8. 11568697 10.1097/00007632-200109010-00011

[CR19] Rubin CI, Atweh GF. The role of stathmin in the regulation of the cell cycle. J Cell Biochem. 2004;93(2):242–50. 15368352 10.1002/jcb.20187

[CR20] Sanada F, et al. IGF Binding Protein-5 Induces Cell Senescence. Front Endocrinol (Lausanne). 2018;9:53. 29515523 10.3389/fendo.2018.00053 PMC5826077

[CR21] Sharma S, McAuley JH. Low Back Pain in Low- and Middle-Income Countries, Part 1: The Problem. J Orthop Sports Phys Ther. 2022;52(5):233–5. 35536248 10.2519/jospt.2022.11145

[CR22] Silwal P, et al. Cellular senescence in intervertebral disc aging and degeneration: molecular mechanisms and potential therapeutic opportunities. Biomolecules. 2023;13(4):686. 37189433 10.3390/biom13040686 PMC10135543

[CR23] Song C, et al. IGFBP5 promotes diabetic kidney disease progression by enhancing PFKFB3-mediated endothelial glycolysis. Cell Death Dis. 2022;13(4):340. 35418167 10.1038/s41419-022-04803-y PMC9007962

[CR24] Soriano-Arroquia A, et al. Age-related changes in miR-143-3p:Igfbp5 interactions affect muscle regeneration. Aging Cell. 2016;15(2):361–9. 26762731 10.1111/acel.12442 PMC4783349

[CR25] Wang F, et al. Aging and age related stresses: a senescence mechanism of intervertebral disc degeneration. Osteoarthritis Cartilage. 2016;24(3):398–408. 26455958 10.1016/j.joca.2015.09.019

[CR26] Wang T, et al. Overexpressed IGFBP5 promotes cell proliferation and inhibits apoptosis of nucleus pulposus derived from rats with disc degeneration through inactivating the ERK/MAPK axis. J Cell Biochem. 2019;120(11):18782–92. 31310371 10.1002/jcb.29191

[CR27] Wang Y, et al. Oxidative stress in intervertebral disc degeneration: Molecular mechanisms, pathogenesis and treatment. Cell Prolif. 2023;56(9):e13448. 36915968 10.1111/cpr.13448 PMC10472537

[CR28] Wen HL, et al. Stathmin, a microtubule-destabilizing protein, is dysregulated in spinal muscular atrophy. Hum Mol Genet. 2010;19(9):1766–78. 20176735 10.1093/hmg/ddq058

[CR29] Wieland OH. The role of hyperglycemia in the pathobiochemistry of diabetes mellitus. Verh Dtsch Ges Inn Med. 1981;87:1–12. 7331415

[CR30] Xu J, et al. Aging, cell senescence, the pathogenesis and targeted therapies of intervertebral disc degeneration. Front Pharmacol. 2023;14:1172920. 37214476 10.3389/fphar.2023.1172920 PMC10196014

[CR31] Zhang H, et al. Time course investigation of intervertebral disc degeneration produced by needle-stab injury of the rat caudal spine: laboratory investigation. J Neurosurg Spine. 2011;15(4):404–13. 21721872 10.3171/2011.5.SPINE10811

[CR32] Zhang GZ, et al. BRD4 Inhibition Suppresses Senescence and Apoptosis of Nucleus Pulposus Cells by Inducing Autophagy during Intervertebral Disc Degeneration: An In Vitro and In Vivo Study. Oxid Med Cell Longev. 2022;2022:9181412. 35308165 10.1155/2022/9181412 PMC8933081

[CR33] Zhang X, et al. Microtubule stabilization promotes the synthesis of type 2 collagen in nucleus pulposus cell by activating hippo-yap pathway. Front Pharmacol. 2023;14:1102318. 36778003 10.3389/fphar.2023.1102318 PMC9909034

[CR34] Zhang GZ, et al. TMT-based proteomics analysis of senescent nucleus pulposus from patients with intervertebral disc degeneration. Int J Mol Sci. 2023;24(17):13236. 37686041 10.3390/ijms241713236 PMC10488253

