# Supplementary material for: STMN1–IGFBP5 axis induces senescence and extracellular matrix degradation in nucleus pulposus cells: In vivo and in vitro insights
Source: Mol Med. 2025 May 3;31:167. doi: 10.1186/s10020-025-01220-7 (PMC12049776; doi:10.1186/s10020-025-01220-7)
Supplement: Supplementary file 1 — Supplementary Material 1. [file 10020_2025_1220_MOESM1_ESM.docx]

**Table S1. Information on NP tissue specimens from included patients.**

| Serial number | Sex | Age (years) | Disease diagnosis | Specimen site | Pfirrmann classification |
| --- | --- | --- | --- | --- | --- |
| 1 | female | 10 | idiopathic scoliosis | T10-T11 | I |
| 2 | male | 12 | idiopathic scoliosis | T12-L1 | I |
| 3 | female | 13 | idiopathic scoliosis | L1-L2 | I |
| 4 | female | 13 | idiopathic scoliosis | T11-T12 | I |
| 5 | female | 14 | idiopathic scoliosis | L1-L2 | I |
| 6 | male | 15 | idiopathic scoliosis | T8-T9 | I |
| 7 | male | 15 | idiopathic scoliosis | L2-L3 | I |
| 8 | female | 17 | idiopathic scoliosis | L3-L4 | II |
| 9 | male | 18 | idiopathic scoliosis | L1-L2 | II |
| 10 | female | 18 | idiopathic scoliosis | L2-L3 | II |
| 11 | female | 43 | lumbar disc herniation | L3-L4 | IV |
| 12 | male | 49 | lumbar disc herniation | L2-L3 | V |
| 13 | male | 53 | lumbar spondylolisthesis | L4-L5 | IV |
| 14 | male | 55 | lumbar disc herniation | L4-L5 | IV |
| 15 | female | 55 | lumbar disc herniation | L5-S1 | IV |
| 16 | female | 57 | lumbar spinal stenosis | L3-L4 | V |
| 17 | male | 59 | lumbar disc herniation | L3-L4 | IV |
| 18 | female | 60 | lumbar disc herniation | L5-S1 | IV |
| 19 | male | 63 | lumbar spondylolisthesis | L3-L4 | IV |
| 20 | female | 65 | lumbar disc herniation | L4-L5 | IV |


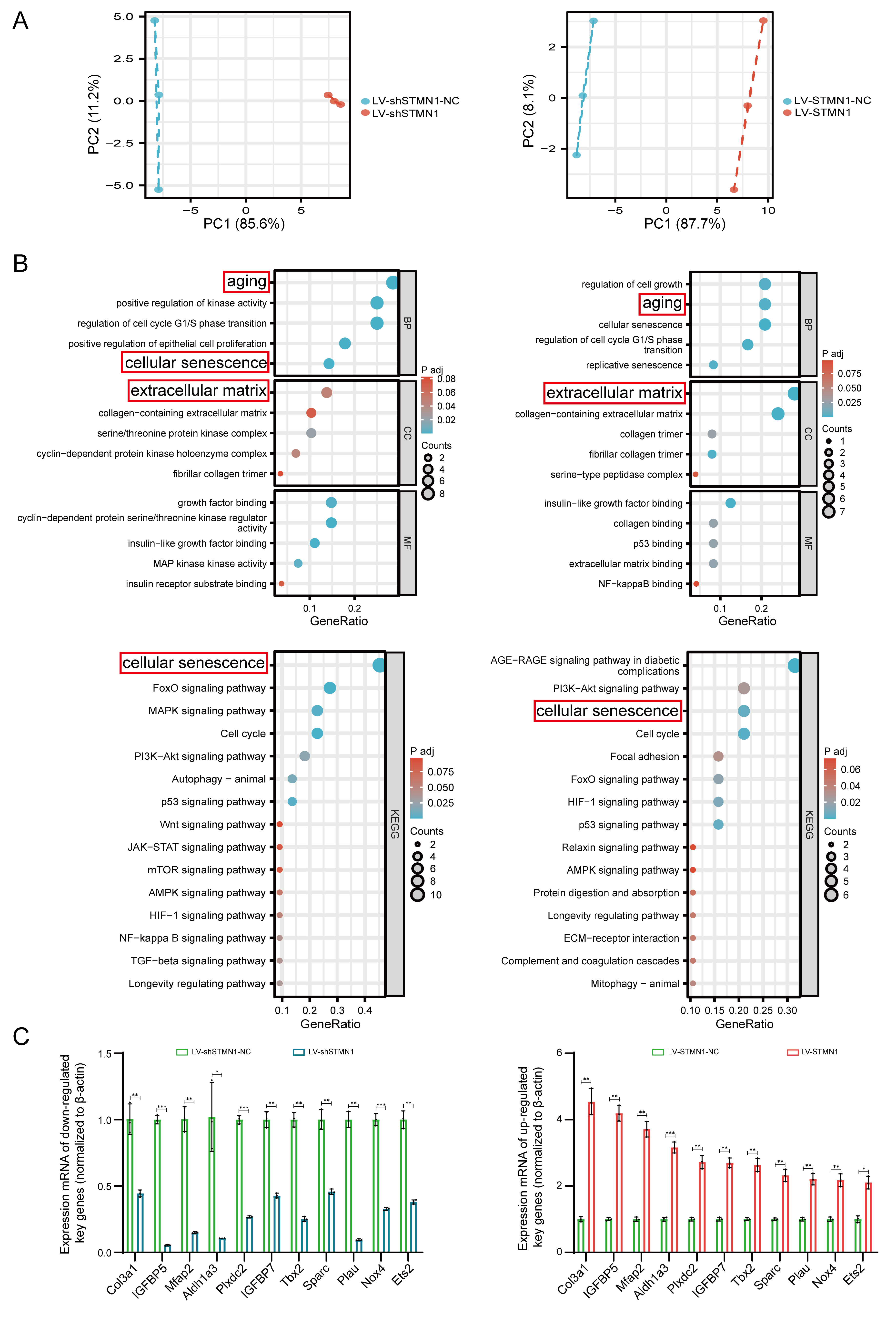


**Fig. S1. Bioinformatics analysis of differential genes and expression validation of mRNAs for key genes**. (A) PCA analysis plots of different intervention groups. (B) GO enrichment and KEGG pathway enrichment of the differential genes. (C) qRT-PCR assay of the 11 key genes for senescence.
